# Calcitriol ameliorates motor deficits and prolongs survival of *Chrne*-deficient mouse, a model for congenital myasthenic syndrome, by inducing Rspo2

**DOI:** 10.1016/j.neurot.2024.e00318

**Published:** 2024-01-16

**Authors:** Bisei Ohkawara, Hiroyuki Tomita, Taro Inoue, Shaochuan Zhang, Shunsuke Kanbara, Hiroyuki Koshimizu, Yuki Miyasaka, Jun-ichi Takeda, Hiroshi Nishiwaki, Hiroaki Nakashima, Mikako Ito, Akio Masuda, Naoki Ishiguro, Tomoo Ogi, Tamio Ohno, Shiro Imagama, Kinji Ohno

**Affiliations:** a Division of Neurogenetics, Center for Neurological Diseases and Cancer, Nagoya University Graduate School of Medicine, Nagoya, Japan; b Department of Orthopedic Surgery, Nagoya University Graduate School of Medicine, Nagoya, Japan; c Department of Genetics, Research Institute of Environmental Medicine (RIeM), Nagoya University, Nagoya, Japan; d Division of Experimental Animals, Nagoya University Graduate School of Medicine, Nagoya, Japan

**Keywords:** Calcitriol, Vitamin D receptor (VDR), Neuromuscular junction, Acetylcholine receptor (AChR) clustering, *Chrne*, and Rspo2

## Abstract

Signal transduction at the neuromuscular junction (NMJ) is compromised in a diverse array of diseases including congenital myasthenic syndromes (CMS). Germline mutations in *CHRNE* encoding the acetylcholine receptor (AChR) ε subunit are the most common cause of CMS. An active form of vitamin D, calcitriol, binds to vitamin D receptor (VDR) and regulates gene expressions. We found that calcitriol enhanced MuSK phosphorylation, AChR clustering, and myotube twitching in co-cultured C2C12 myotubes and NSC34 motor neurons. RNA-seq analysis of co-cultured cells showed that calcitriol increased the expressions of *Rspo2*, *Rapsn,* and *Dusp6*. ChIP-seq of VDR revealed that VDR binds to a region approximately 15 ​kbp upstream to *Rspo2*. Biallelic deletion of the VDR-binding site of *Rspo2* by CRISPR/Cas9 in C2C12 myoblasts/myotubes nullified the calcitriol-mediated induction of *Rspo2* expression and MuSK phosphorylation. We generated *Chrne* knockout (*Chrne* KO) mouse by CRISPR/Cas9. Intraperitoneal administration of calcitriol markedly increased the number of AChR clusters, as well as the area, the intensity, and the number of synaptophysin-positive synaptic vesicles, in *Chrne* KO mice. In addition, calcitriol ameliorated motor deficits and prolonged survival of *Chrne* KO mice. In the skeletal muscle, calcitriol increased the gene expressions of *Rspo2*, *Rapsn,* and *Dusp6*. We propose that calcitriol is a potential therapeutic agent for CMS and other diseases with defective neuromuscular signal transmission.

## Graphical abstract

**Figure:**
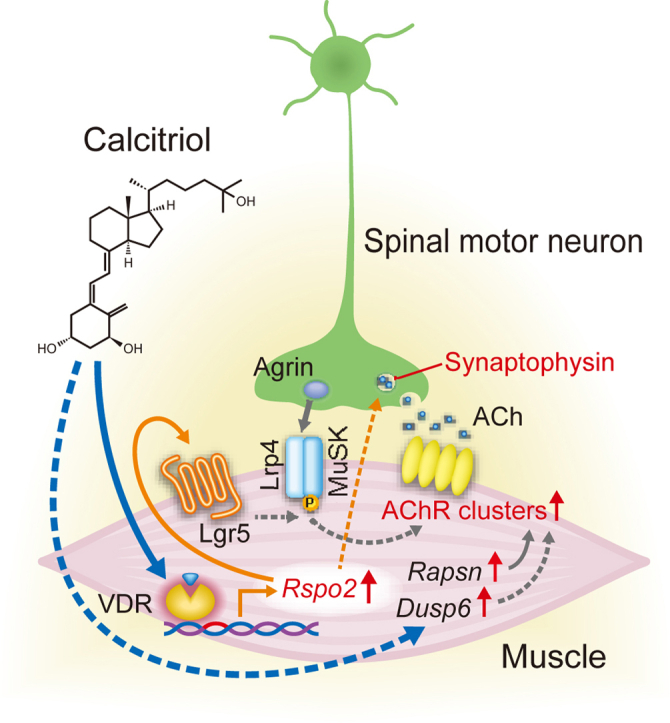
An active form of vitamin D, calcitriol, directly enhances the expression of *Rspo2* by binding to the vitamin D receptor (VDR). Rspo2 binds to Lgr5 and enhances the agrin-LRP4-MuSK signaling pathway to facilitate the clustering of acetylcholine receptor (AChR) clusters. Calcitriol markedly increases synaptic vesicles likely via Rspo2. Rspo2 also indirectly enhances the expressions of *Rapsn* and *Dups6* to facilitate AChR clustering. Solid and broken lines indicate direct and indirect effects, respectively.

## Introduction

Calcitriol, 1,25-dihydroxycholecalciferol, is an active form of vitamin D. Vitamin D is primarily produced in the skin when it is exposed to ultraviolet rays, and is activated by two enzymatic hydroxylation steps in the liver and the kidney [1]. Calcitriol is a fat-soluble vitamin that binds to and activates a nuclear receptor, vitamin D receptor (VDR), and exerts physiological effects by enhancing transcription of target genes [2]. Calcitriol stimulates the release of Ca^2+^ by upregulating expressions of *Rankl2*, *Fgf23*, *Vdr,* and others in osteoblasts, and the released Ca^2+^ activates osteoclasts [3]. In addition, calcitriol promotes absorption of dietary Ca^2+^ from the gastrointestinal tract and increases renal tubular reabsorption of Ca^2+^ to increase Ca^2+^ concentration in the blood. Lack of calcitriol provokes Ca^2+^ deficiency, which subsequently causes rickets in children and osteomalacia in adults [4]. Mortality of elderly people is elevated at both high and low levels of calcifediol, a precursor for calcitriol, compared to that at a moderate level [5]. Calcitriol ameliorates hypertension [6] and cancer [7], and also works on the brain development and functions [8]. In addition, calcitriol is one of candidate repositioning drugs for autism spectrum disorder [9], influenza [10], COVID-19 [11], fungal infections [12], and ovarian cancer [13], although the pharmacological mechanisms remain elusive except for ovarian cancer, in which the suppression of Smad signaling by calcitriol exerted its antitumor effect [13]. In contrast to the effect of calcitriol on diseases, meta-analyses showed that the benefit of vitamin D supplements to musculoskeletal performances in healthy subjects is not observed or marginal [[14], [15], [16]]. Nevertheless, knockout of *Vdr* in mice nullified the exercise-induced enhancement of locomotive ability [17]. A recent study showed that calcitriol enhanced rapsyn expression and agrin-induced acetylcholine receptor (AChR) clustering in cultured myotubes [18].

The neuromuscular junction (NMJ) is a synapse between the spinal motor neuron (SMN) and the skeletal muscle. At the NMJ, the contraction of myofibers is controlled by the neurotransmitter acetylcholine (ACh) released from the motor nerve terminal [19]. To ensure efficient neuromuscular signal transmission, a combination of secreted molecules acts in a concerted manner to regulate pre- and postsynaptic organization of the NMJ [20]. At the postsynaptic region of the NMJ, SMN-derived agrin binds to Lrp4 and phosphorylates MuSK [[21], [22], [23]], which leads to rapid tyrosine phosphorylation on the AChR β subunit [24] and facilitates AChR clustering anchored to subsynaptic scaffolding proteins including rapsyn [25]. In addition, we previously showed that a secreted protein Rspo2 enhances the agrin-Lrp4-MuSK signaling pathway and facilitates postsynaptic AChR clustering and the NMJ formation [20,26]. Rspo2 is secreted from both the SMNs and the skeletal muscles. SMN-specific rescue of Rspo2 in *Rspo2* KO mice mostly ameliorated the defects in AChR clustering and NMJ formation, whereas skeletal muscle-specific rescue of Rspo2 scarcely ameliorated them, suggesting that SMN-derived Rspo2 is critical for the enhancement of the agrin-Lrp4-MuSK pathway [27]. At the presynaptic nerve terminal of SMN, retrograde signals from the postsynaptic region by *Lrp4* [28,29], *Slit2* [30], *Rspo2* [26,27] induce the clustering of synaptic vesicles and active zones.

Signal transduction at the NMJ is compromised in a diverse array of diseases including congenital myasthenic syndromes (CMS) [31,32]. CMS are heterogeneous disorders in which the safety margin of neuromuscular transmission is compromised by one or more specific mechanisms. Approximately one-half of CMS stem from mutations in the AChR subunit genes (*CHRNA1*, *CHRNB1*, *CHNRD*, and *CHRNE*) [31,32]. The adult-type AChR ε subunit (*CHRNE*) appears after birth to substitute for the embryonic AChR γ subunit (*CHRNG*), and is exclusively expressed at the motor endplate of the NMJ [33]. *Chrne* knockout (KO) mice appear externally normal for the first few weeks after birth, because of the compensatory expression of the γ subunit [34,35]. However, at around 4–5 weeks of age, *Chrne* KO mice become noticeably weaker and less active than *Chrne* heterozygous littermates [34,35]. *Chrne* KO mice remain viable until 7–12 weeks of age, but most of them die at 8–12 weeks. Thus, *Chrne* KO mice serve as a model for a severe and delayed-onset CMS.

Here we show that calcitriol enhances the expression of Rspo2 via a VDR-biding site for *Rspo2* and ameliorates abnormal NMJ phenotypes in *Chrne* KO mice.

## Materials and Methods

### Single culture of C2C12 myotubes

**Fig. 1:**
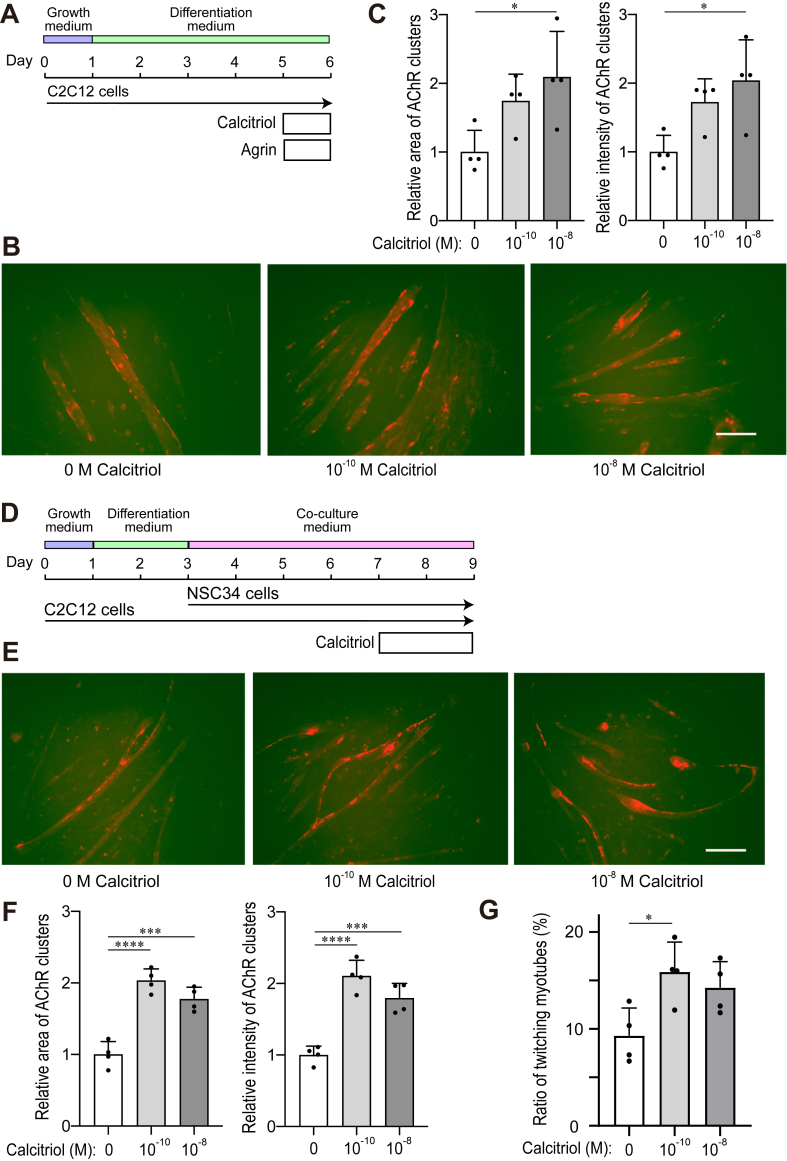
Calcitriol enhances AChR clustering in C2C12 myotubes co-cultured with NSC34 ​cells **(A)** Culturing protocol of C2C12 myoblasts/myotubes with 10 ​ng/ml agrin and 10^−10^ or 10^−8^ ​M calcitriol for 24 ​h ​for (**B** and **C**). **(B, E)** Representative AChR clusters stained with α-bungarotoxin (red) on C2C12 myotubes with the indicated concentrations of calcitriol. Bar ​= ​10 ​μm. **(C, F)** Total area and total intensity of AChR clusters were blindly measured using MetaMorph software, and values were normalized to that without calcitriol. Mean and SD are indicated (*n* ​= ​6 visual fields per well ​× ​4 wells). ∗*p* ​< ​0.05, ∗∗∗*p* ​< ​0.001, and ∗∗∗∗*p* ​< ​0.0001 by one-way ANOVA followed by Tukey’s posthoc test. **(D)** Co-culturing protocol of C2C12 myoblasts/myotubes and NSC34 motor neurons with 10^−10^ or 10^−8^ ​M calcitriol for (**E**, **F**, and **G**). **(G)** Ratio of twitching myotubes in four wells was blindly counted. Mean and SD are plotted. ∗*p* ​< ​0.05 by one-way ANOVA followed by Dunnett’s posthoc test compared to 0 ​M calcitriol.

C2C12 immortalized mouse myoblasts were purchased from ATCC (CRL-1772). C2C12 ​cells were seeded at 100 ​cells/mm^2^ on a 12-well or 6-well plate coated with 50 ​μg/ml collagen 1 (Corning, 354236), and cultured in growth medium (GM) constituted of DMEM, 10 ​% fetal bovine serum, and 1 ​% Pen-Strep (Thermo Fisher Scientific, 15140-122). One day after the passage (day 1), the medium was changed to differentiation medium (DM) constituted of DMEM, 2 ​% horse serum (Gibco, 1998112), and 1 ​% Pen-Strep (Fig. 1A). On day 5, calcitriol with or without agrin (5 or 10 ​ng/ml, R&D Systems, AZH1216091) was added for 16 or 24 ​h. For blocking protein synthesis, 10 ​μg/ml cycloheximide (Fujifilm Wako Pure Chemical, 037-20991) was added before adding calcitriol and agrin. On day 6, cells were fixed to measure AChR clusters or harvested to quantify gene expressions. Cell images were taken with an Olympus IX71 microscope. To examine MuSK phosphorylation in C2C12 myotubes, calcitriol and agrin were added 16 ​h and 1 ​h before harvesting cells, respectively. The concentrations of calcitriol were 10^−10^ or 10^−8^ ​M.

### Co-culture of C2C12 myotubes and NSC34 neuronal cells

A co-culture plate was coated with 50 ​μg/mL collagen I. C2C12 ​cells (100 ​cells/mm^2^) were cultured in GM for 1 day (from days 0–1) and then cultured in DM for 2 additional days (from days 1–3) (Fig. 1D). On day 3, NSC34 ​cells were seeded at 50 ​cells/mm^2^ on the partially differentiated C2C12 ​cells in co-culture medium (CCM) constituted of DMEM/F12, 0.5 ​% horse serum, 1 ​% non-essential amino acid (MP Biomedicals, 1681049), and 1 ​% Pen-Strep. The cells were differentiated into either myotubes or neurite-elongated motor neurons for 4 days (days 3–7) and then cultured with or without 10^−10^ or 10^−8^ ​M calcitriol for 2 days (days 7–9). CCM was changed every other day up to day 9. On day 9, AChR clustering, myotube twitching, and mRNA expressions by RNA-seq were quantitated, as previously described [36].

### Staining of AChR clusters in C2C12 myotubes

C2C12 myotubes were incubated with 10 ​μg/ml Alexa594-conjugated α-bungarotoxin (Invitrogen, 1938422). The cells were then fixed in 4 ​% paraformaldehyde (PFA) for 15 ​min. Before and after fixation, the cells were washed with phosphate buffered saline (PBS) three times. For peripherin staining in addition to α-bungarotoxin staining, the cells were treated with PBS containing 2 ​% goat serum (Fujifilm Wako Pure Chemical, WDR2410) and 0.1 ​% Triton X-100 for 1 ​h. After washing with PBS, the cells were incubated with rabbit polyclonal anti-peripherin antibody (1:300, Millipore, 2972430) overnight at 4 ​°C, and with Alexa488-conjugated goat anti-rabbit IgG (1:100, Invitrogen, SH251139) for 1 ​h at room temperature. The staining was observed with an IX71 fluorescence microscope (Olympus) and analyzed by CellSens software (Olympus). The total area and the total intensity of AChR clusters were blindly evaluated using MetaMorph software (Molecular Devices).

### Evaluation of myotube twitching

C2C12 myotubes were co-cultured with NSC34 ​cells on a 12-well plate with 10^−10^ or 10^−8^ ​M calcitriol, as stated above (Fig. 1D). On day 9, the total number of twitching myotubes was blindly counted for 5 ​min per visual field in 20 visual fields per well ​× ​4 wells under an IX71 fluorescence microscope (Olympus).

### High-throughput RNA sequencing (RNA-Seq)

RNA from co-cultured C2C12 myotubes and NSC34 motor neurons in three independent wells of 6-well plates were subjected to RNA-seq. Total RNA was isolated with the QuickGene RNA cultured cell kit (Kurabo) using QuickGene-800 (Kurabo). Total RNA was harvested from the triceps brachii muscle of wild-type and *Chrne* KO mice using Trizol Reagent (Thermo Fisher Scientific) followed by RNA isolation with the RNeasy Plus Mini Kit (Qiagen, Tokyo, Japan) according to the manufacturer's instructions. Quality of the RNA samples was checked using both an Agilent TapeStation and an Agilent 2100 Bioanalyzer with the RNA 6000 Nano Kit, and the following thresholds were applied: quantity >100 ​ng, concentration >1 ​ng/μl, no contamination of DNA, no degradation of RNA confirmed by rRNA peaks (18S, 28S), and RNA integrity number (RIN) ​> ​7.0. A sequencing library for RNA-seq was prepared using the TruSeq Stranded mRNA kit (Illumina), and the library was read on Illumina NovaSeq 6000 (150 bp paired-end reads) at Macrogen. Transcripts per million (TPM) of each gene was calculated by RSEM version 1.3.1 with default parameters. The RNA-seq analysis was triplicated. The RNA-seq data were deposited in the DNA Data Bank of Japan (DDBJ) with the accession number of DRA016803.

### Gene set enrichment analysis (GSEA) of RNA-seq of co-cultured C2C12 and NSC34 ​cells

Expressions of each gene in co-cultured C2C12 and NSC34 ​cells with and without 10^−10^ ​M calcitriol were compared. A threshold of fold-change (FC) was arbitrarily set to log_2_(FC) ​< ​−4.5 or log_2_(FC) ​> ​4.5. In addition, a threshold of false discovery rate (FDR) was arbitrarily set to -log_10_(FDR) ​> ​1.5. Pathways were analyzed with GSEA [37] using the Reactome pathway database comprised of 1554 gene sets [38].

### Quantitative RT-PCR (qRT-PCR) analysis

Total RNA in cultured cells was isolated with the QuickGene RNA cultured cell kit (Kurabo) using QuickGene-800 (Kurabo). Total RNA in diaphragm was purified by TRIzol RNA isolation reagent (Thermo Fisher Scientific, 15596026) and further by RNeasy Mini Kit (Qiagen, 74104) following the manufacturer’s instructions. First-strand cDNA was synthesized with ReverTra Ace (Toyobo). mRNA expression levels were quantified using LightCycler 480 Real-Time PCR (Roche) and SYBR Green (Takara), and were normalized to that of β2 microglobulin (*B2m*). The primer sequences are shown in Suppl. Table S1.

### Evaluation of ChIP-seq dataset of osteocytic IDG-SW3 cells

ChIP-seq dataset with antibody against VDR with or without 10^−7^ ​M calcitriol in IDG-SW3 bone cells was obtained from the Gene Expression Omnibus (GSE54784) [39]. ChIP-seq peaks were inspected with Integrative Genomics Viewer 2.9.4.

### Establishment of C2C12 ​cells lacking the VDR-binding site in the distal promoter region of *Rspo2*

Simultaneous targeting of two sites using two distinct guide RNA (gRNA) sequences results in the deletion of the intervening sequence by non-homologous end joining. To delete the VDR-binding site in the distal promoter region of *Rspo2*, gRNAs flanking the VDR-binding ChIP-seq peak were designed using CHOPCHOP (https://chopchop.cbu.uib.no/). gRNAs were 5′-ATACAGTCAAGCCAGAATGCAGG-3′ at chr15: 43,179,481–43,179,503 (GRCm38/mm10) and 5′-AGTGACATTCACAGCTACTGTGG-3′ at chr15: 43,180,143–43,180,165 (GRCm38/mm10). gRNAs and the Cas9 protein (Thermo Fisher Scientific) were introduced into C2C12 cells by electroporation [40]. Electroporated cells were cultured in two dishes. At 48 ​h after electroporation, DNA was extracted from a single dish, and the presence of deleted alleles in a fraction of cells were confirmed by PCR spanning the two gRNAs. Next, 200–400 ​cells on another dish were plated on a 10-cm dish for isolation of single colonies for 7–10 days. Single colonies were picked up, and further expanded in 48-well plates.

### Generation of *Chrne* KO mouse line

All mouse studies were approved by the Animal Care and Use Committee of the Nagoya University and were performed in accordance with relevant guidelines. To delete exons 2 to 5 of *Chrne*, C57BL/6J zygotes were microinjected with Cas9 protein (New England Biolabs, M0646 ​M) and gRNAs (*Chrne*_crRNA_g70618969-70618991 on GRCm38/mm10, 5′-TAAACACAGGAGGGGTGCAATGG-3′ and *Chrne*_crRNA_g70617987-70618009 on GRCm38/mm10, 5′-GTGTAGGTTTGCAGTAGGCGTGG-3′). The deletions introduced by the CRISPR/Cas9 system were confirmed by Sanger sequencing with PCR primers on exons 1, 2, and 6 (Ex1F, 5′-CCCTGCTTCTCCTGACACTCTTTG-3’; Ex2R, 5′-GTGACCTTGAGGGTGATGGT-3’; and Ex6R, 5′-TGAACTCCACCTCCTCAGCATTGTAG-3′). Possible off-target sites were predicted by the CRISPOR website. The top five high-scored regions for each set of gRNAs were also analyzed by Sanger sequencing, and no artificial mutations were observed. Heterozygous mice were then crossed with C57BL/6 mice to give rise to heterozygous and finally homozygous mutants.

### Mouse studies

Mice were housed at 23 ​°C under a 12-h light/dark cycle with *ad labium* access to diet and water. Six-week-old male or female *Chrne*-deficient mouse were randomly divided into two groups: a control group administered with 1 ​% ethanol and a treated group administered with 0.016–1.6 ​μg/kg/day calcitriol (hydrochloride form of a racemic mixture, MP Biochemicals, 155341) in 1 ​% ethanol. The drug was intraperitoneally administrated with syringe (Termo, SS10M2713) every day for 2–6 weeks starting from age 6 weeks, because *Chrne* KO mice started to die around age 7 weeks. For analyzing the mouse survival, calcitriol was intraperitoneally administrated every other day. Body weights of mice were measured every day.

Motor activities of vehicle- and calcitriol-treated mice were examined by the rota-rod test and the voluntary motor activity test at age 6, 7, and 8 weeks. The rota-rod (Ugo Basile) stayed at 5 ​rpm for 5 ​s, and was linearly accelerated from 5 to 50 ​rpm over 240 ​s. A timer was started when acceleration started and was counted up to 500 ​s. A mouse was acclimated to the rota-rod test once, and was tested four times at an interval of 15 ​min. Voluntary motor activity was evaluated by an IR actinometer (Harvard Apparatus, LE 8825 Motor Activity Monitor). Each mouse was placed in a cage implemented with a grid of infrared beams (24.0 ​cm diameter and 17.0 ​cm width), in which the number and the frequency of crossing the infrared beams per hour were counted. The mouse was acclimatized to the cage one day before the measurement.

At 2 weeks after starting calcitriol administration, the quadriceps femoris, the gastrocnemius, and the tibialis anterior in both hindlimbs of vehicle- and calcitriol-treated mice were dissected, and the wet weights were measured. Left diaphragm was used for qRT-PCR analysis. Right diaphragm was fixed with PFA for 2 ​h at 4 ​°C for histological analysis.

Kaplan-Meier curves were plotted for survival rate analysis, and a log-rank test was performed for comparison.

### Diaphragm staining

The right diaphragms of wild-type and *Chrne* KO mice at 8 weeks of age were fixed in 2 ​% PFA in PBS for 4 ​h at 4 ​°C and rinsed with PBS. After removing the connective tissue, the muscles were permeabilized with 0.5 ​% Triton X-100 in PBS for 10 ​min and then incubated with anti-synaptophysin antibody (1:100, Invitrogen, 180130) overnight. After washing, the sections were incubated with α-bungarotoxin conjugated with Alexa 564 (1:100, Invitrogen, 1938422) and anti-mouse IgG conjugated with Alexa 488 (1:500, Invitrogen). Fluorescence images were obtained using a Nikon A1Rsi confocal microscope for high magnification images of the NMJ (*n* ​= ​40–50 images for each right diaphragm ​× ​four diaphragms). The area, intensities, and numbers of AChR signals and synaptophysin signals were automatically quantified using MetaMorph software (Molecular Devices). The quantifications were totally blinded.

### Statistical analysis

Data are expressed as mean and standard deviation (SD). Statistical analyses were carried out using either unpaired Student’s *t*-test, one-way or two-way ANOVA with Dunnett’s, Tukey’s, or Sidak’s post-hoc test using GraphPad Prism ver. 8.4.3. Default posthoc tests of the Prism software were applied. *P* values less than 0.05 were considered significant.

## Results

### Calcitriol induces *Rapsn* and *Rspo2* expression in co-cultured C2C12 myotubes and NSC34 neuronal cells

We first sought for an optimal concentration of calcitriol to enhance agrin-induced AChR clustering on day 6 of myotube differentiation of C2C12 ​cells. We found that 10^−9^ ​M calcitriol was more potent than 10^−12^ ​M and that 10^−6^ ​M exerted toxic effects on the cells (Suppl. Fig. S1). We next compared 10^−10^ and 10^−8^ ​M calcitriol and found that both concentrations similarly enhanced agrin-induced AChR clustering (Fig. 1A–C). We recently showed that co-culture of C2C12 myotubes and neurite-elongating NSC34 ​cells made a small fraction of AChR clusters of C2C12 myotubes juxtaposed to peripherin-positive neurites of NSC34 ​cells [36]. We next examined the effect of calcitriol on co-cultured cells. The area and intensity of AChR clusters were increased by 10^−10^ and 10^−8^ ​M calcitriol in co-cultured C2C12 myotubes on day 9 (Fig. 1D–F). Calcitriol (10^−10^ ​M) also increased the number of twitching myotubes in co-cultured C2C12 myotubes (Fig. 1G). In both assays, 10^−10^ and 10^−8^ ​M calcitriol showed similar effects, and 10^−10^ ​M calcitriol was used in the following cell studies.

**Fig. 2:**
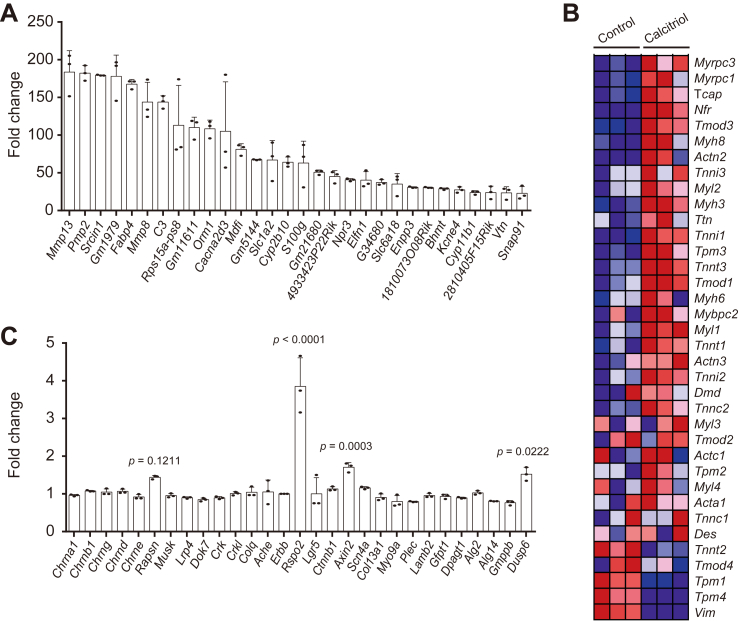
RNA-seq analysis of co-cultured C2C12 myotubes and NSC34 motor neurons with or without calcitriol (*n* ​= ​3 wells each) **(A)** Top 30 genes increased by 10^−10^ ​M calcitriol. Mean and SD are plotted. **(B)** Heatmap of genes in the Reactome pathway “striated muscle contraction”. The heatmap was automatically generated by GSEA. For each gene, the lowest and the highest values are shown in dark blue and dark red, respectively. See Suppl. Fig. S2B for the enrichment plot of “striated muscle contraction”. **(C)** Representative upregulated genes that are mutated in CMS or highly expressed at the NMJ [43,53]. Mean and SD are plotted. *P*-values by two-way ANOVA followed by Sidak’s posthoc test are indicted for the top four genes. Two-way ANOVA compared gene expressions between control- and calcitriol-treated co-cultured cells.

To dissect the underlying molecular mechanism, we performed RNA-seq analysis of co-cultured cells with or without 10^−10^ ​M calcitriol (Suppl. Fig. S2A). Calcitriol binds to its nuclear receptor, VDR, and increases the expressions of *Mmp13* and *Enpp3* in osteoblastic MC3T3-E1 cells [41] and differentiated osteoblasts [39,42], respectively, both of which are representative marker genes of calcitriol. RNA-seq analysis showed that 10^−10^ ​M calcitriol increased the expressions of *Mmp13* and *Enpp3* in co-cultured cells (Fig. 2A). GSEA analysis showed that calcitriol did not significantly increase any Reactome pathway (Suppl. Table S1). Although statistical significance was not observed in the false discovery rate (FDR), “striated muscle contraction” was ranked tenth with unadjusted *p* ​= ​0.033. Indeed, genes in the “striated muscle contraction” were increased by calcitriol (Fig. 2B, Supplementary Fig. S2B). GSEA analysis also showed that calcitriol suppressed 75 of the 1554 pathways in Reactome with FDR <0.05 (Suppl. Table S2). Most of the significantly decreased pathways were related to cell divisions. Thus, calcitriol was likely to have switched the C2C12 and NSC34 ​cells from cell growth to differentiation.

Among the genes that are mutated in CMS or highly expressed at the NMJ [43], calcitriol tended to increase *Rapsn,* as previously reported [18] (Fig. 2C). Calcitriol also increased *Rspo2*, *Axin2*, and *Dusp6*. Rspo2 [26] and rapsyn [44] are upstream and downstream of agrin-induced MuSK phosphorylation, respectively, and both are essential for AChR clustering. The other calcitriol-induced genes, *Axin2* [45] and *Dusp6* [43], are also expressed at the NMJ, but their roles in MuSK phosphorylation and AChR clustering remain unknown.

### Calcitriol directly induces *Rspo2* expression by enhancing VDR binding to the distal promoter region of *Rspo2* in C2C12 myotubes

**Fig. 3:**
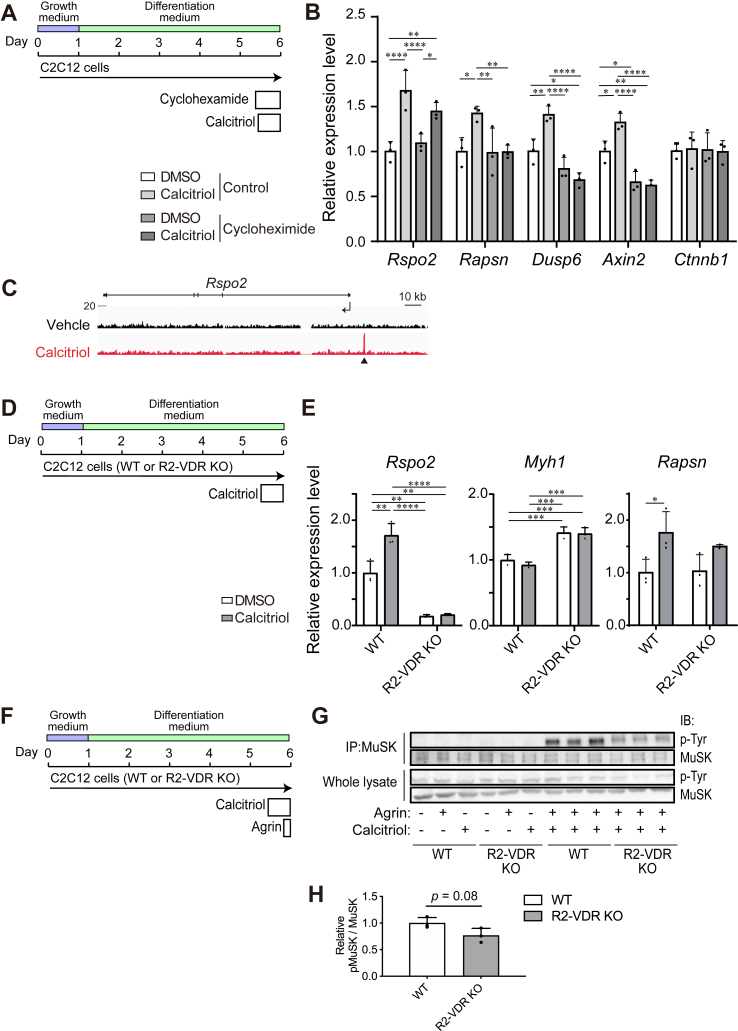
Calcitriol enhances *Rspo2* expression via distal promoter region of *Rspo2* and enhances MuSK phosphorylation **(A)** Culturing protocol of C2C12 myotubes with cycloheximide for 18 ​h and 10^−10^ ​M calcitriol for 16 ​h. (**B, E**) qRT-PCR of indicated genes. Gene expression was normalized to that of *B2m* and then to the ratio of DMSO without cycloheximide **(B)** or DMSO-treated WT C2C12 ​cells **(E)**. Mean and SD are indicated (*n* ​= ​3 wells each). ∗*p* ​< ​0.05, ∗∗*p* ​< ​0.01, ∗∗∗*p* ​< ​0.001, and ∗∗∗∗*p* ​< ​0.0001 by one-way ANOVA followed by Tukey’s posthoc test. **(C)** ChIP-seq tracks of VDR for *Rspo2* normalized to 10^7^ tags in control- and calcitriol-treated IDG-SW3 mouse osteocytic cells that were differentiated for 35 days [39]. An arrowhead points to the VDR-binding site ∼15 ​kbp upstream to *Rspo2*. **(D)** Culturing protocol of wild-type (WT) and genetically engineered C2C12 ​cells lacking the VDR-binding site at the distal promoter region of *Rspo2* (R2-VDR KO) with 10^−10^ ​M calcitriol for 16 ​h. **(F)** Culturing protocol of WT and R2-VDR KO C2C12 myotubes with 5 ​ng/ml agrin for 1 ​h and 10^−10^ ​M calcitriol for 16 ​h. (**G**) Total MuSK was immunoprecipitated (IP) with an anti-MuSK antibody, and phosphorylated MuSK was immunoblotted with an anti-phosphotyrosine (p-Tyr) antibody. Note that the concentration of agrin was reduced to 5 ​ng/ml so that MuSK phosphorylation was marginally induced by agrin alone. (**H**) Signal intensities of phosphorylated MuSK were normalized for total MuSK in agrin- and calcitriol-treated cells, and then for the ratio of wild-type (WT) C2C12 ​cells. Mean and SD are indicated (*n* ​= ​3 lanes each). Statistical significance was calculated by unpaired Student’s *t*-test.

We next examined which gene(s) were directly induced by calcitriol. To this end, we blocked protein synthesis with cycloheximide before adding calcitriol and analyzed gene expressions. Even when protein synthesis was blocked for 18 ​h, calcitriol still enhanced the expression of *Rspo2* but not of *Rapsn*, *Axin2*, or *Dusp6* in C2C12 myotubes (Fig. 3A, B). Thus, *Rspo2* is likely to be a direct target of calcitriol, whereas *Rapsn*, *Axin2*, or *Dusp6* are likely to be secondary targets.

Inspection of the ChIP-seq dataset of VDR in calcitriol-treated osteoblasts [39] revealed that calcitriol triggers the binding of VDR to a region approximately 15 ​kbp upstream to *Rspo2* (Fig. 3C). Rspo2 is expressed in both SMNs and skeletal muscle. Although SMN-derived Rspo2 rather than skeletal muscle-derived Rspo2 enhanced the agrin-Lrp4-MuSK pathway [27], biallelic deletion of the VDR-binding site in both NSC34 and C2C12 ​cells was technically challenging. We thus deleted the VDR-binding site upstream to *Rspo2* only in C2C12 ​cells with CRISPR/Cas9 system. We obtained a single colony of C2C12 ​cells with a 633-bp deletion on one allele and a 631-bp deletion on another allele (R2-VDR KO C2C12 ​cells) (Suppl. Fig. S3A). When the VDR-binding site was deleted, the *Rspo2* expression was markedly attenuated and the *Myh1* expression, a differentiation marker, was upregulated (Fig. 3D, E). In addition, in R2-VDR KO C2C12 ​cells, calcitriol failed to induce the *Rspo2* expression, whereas the calcitriol-induced expression of *Rapsn* was not affected (Fig. 3D, E). We next analyzed the effect of calcitriol-induced expression of *Rspo2* on MuSK phosphorylation and AChR clustering in C2C12 myotubes. In wild-type C2C12 myotubes, agrin and calcitriol co-operatively enhanced MuSK phosphorylation (Fig. 3FG) and AChR clustering (Suppl. Fig. S3C). In R2-VDR KO C2C12 myotubes, agrin-induced MuSK phosphorylation tended to be reduced (Fig. 3F, G), whereas agrin-induced AChR clustering was not affected (Suppl. Fig. S3C). Calcitriol also induced NMJ-enriched genes including *Rapsn*, *Dusp6*, and *Axin2* not via *Rspo2*, which was likely to account for the preserved AChR clustering activity in the lack of the VDR-binding site of *Rspo2*.

### Calcitriol ameliorates dysregulated presynaptic region of the NMJ and motor activity in *Chrne*-deficient (*Chrne* KO) mouse

To examine the *in vivo* effects of calcitriol, we generated *Chrne* KO mice by the CRISPR/Cas9 system. *Chrne* heterozygous mice showed no apparent pathological phenotypes, as previously reported by others [34,35]. The homozygous mice appeared normal for the first few weeks after birth, but at around 1 month of age they became noticeably weaker and less active than their littermates. The mice also ceased to gain weight at this time and developed a characteristic whistling noise accentuated during exercise or excitation. Some mutant mice died around 7 weeks of age, most died at 9–10 weeks, and none lived past 12 weeks. All these phenotypes were similar to previous reports of *Chrne*-mutant mice [34,35]. Thus, the *Chrne* KO mice modeled for a severe and delayed-onset CMS.

**Fig. 4:**
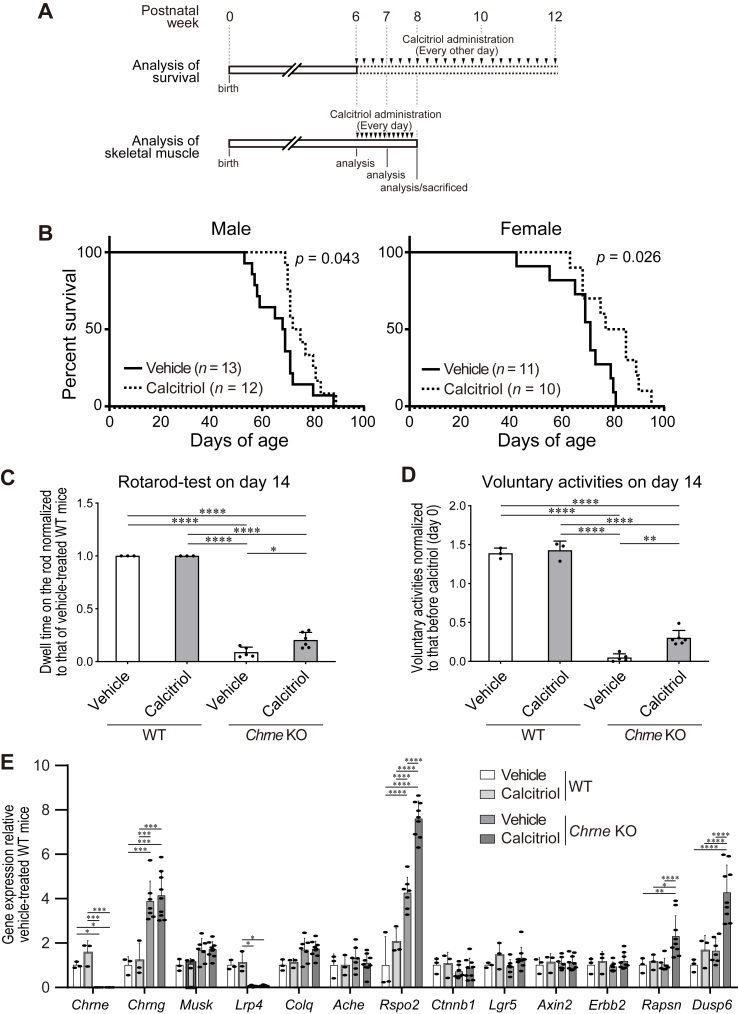
Calcitriol improves motor deficits and increases gene expressions of *Rspo2*, *Rapsn*, and *Dusp6* in tibialis anterior muscle of *Chrne* KO mouse. **(A)** Protocols of intraperitoneal administration of 1 ​% ethanol (vehicle) and 0.016 ​μg/kg/day calcitriol for *Chrne* KO mice starting from 6 weeks of age. For the analysis of mouse survival, calcitriol was administered every other day until the mouse died. For the analysis of the skeletal muscle, calcitriol was administered every day for 2 weeks. **(B)** Survival rate of *Chrne* KO male and female mice with or without calcitriol treatment (0.016 ​μg/kg/day). The survival rate of calcitriol-treated mice was significantly longer than that of vehicle-treated mice in both males (*p* ​= ​0.043) and females (*p* ​= ​0.026) by log-rank test. **(C)** Endurance time on a rota-rod of wild-type (WT) and *Chrne* KO mice with or without calcitriol for 2 weeks. Mean and SD are indicated. ∗*p* ​< ​0.05 and ∗∗∗∗*p* ​< ​0.0001 by one-way ANOVA followed by Tukey’ s posthoc test. The mice were observed up to 500 ​s on the rotarod. The actual endurance time of each mouse is indicated in Suppl. Fig. S4DF. **(D)** Voluntary motor activities measured by an IR Actinometer of wild-type (WT) and *Chrne* KO mice with or without calcitriol for 2 weeks. Mean and SD are indicated. ∗∗*p* ​< ​0.01 and ∗∗∗∗*p* ​< ​0.0001 by one-way ANOVA followed by Tukey’ s posthoc test. The actual number of crossing beams per hour of each mouse is indicated in Suppl. Fig. S4EG. **(E)** qRT-PCR of genes related to NMJ and muscle differentiation. Gene expression was normalized to that of *B2m* and then to the ratio of vehicle-treated wild-type (WT) mice. Mean and SD are indicated (*n* ​= ​12 wells each). ∗*p* ​< ​0.05, ∗∗*p* ​< ​0.01, ∗∗∗*p* ​< ​0.001, and ∗∗∗∗*p* ​< ​0.0001 by one-way ANOVA followed by Tukey’s posthoc test.

*Chrne* KO mice were intraperitoneally administered with calcitriol from 6 weeks of age. We first examined an appropriate concentration of calcitriol for *Chrne* KO mice by intraperitoneally administering 0.016, 0.16, and 1.6 ​μg/kg/day of calcitriol every day for 2 weeks from age 6 weeks. We found that 0.016 ​μg/kg/day of calcitriol did not decrease the body weight, but the other concentrations tended to decrease it (Suppl. Fig. S4A). We thus used 0.016 ​μg/kg/day calcitriol in the following studies, which was equivalent to the conventional dose of 1.0 ​μg/day calcitriol for patients with osteoporosis and hypoparathyroidism. Administration of calcitriol every other day from 6 weeks of age increased the survival of both male and female *Chrne* KO mice (Fig. 4AB). Administration of calcitriol every day from 6 weeks of age for 2 weeks did not change the body and muscle weights (Suppl. Fig. S4BC), but increased endurance exercise performances on a rotarod (Fig. 4C) and voluntary motor activities (Fig. 4D) compared to those of vehicle-treated *Chrne* KO mice. The statistical differences in motor activities were observed on day 14 but not on day 7 (Suppl. Fig. S4FG). Taken together, calcitriol treatment ameliorated forced and voluntary motor activities, and prolonged the survival of *Chrne* KO mice.

**Fig. 5:**
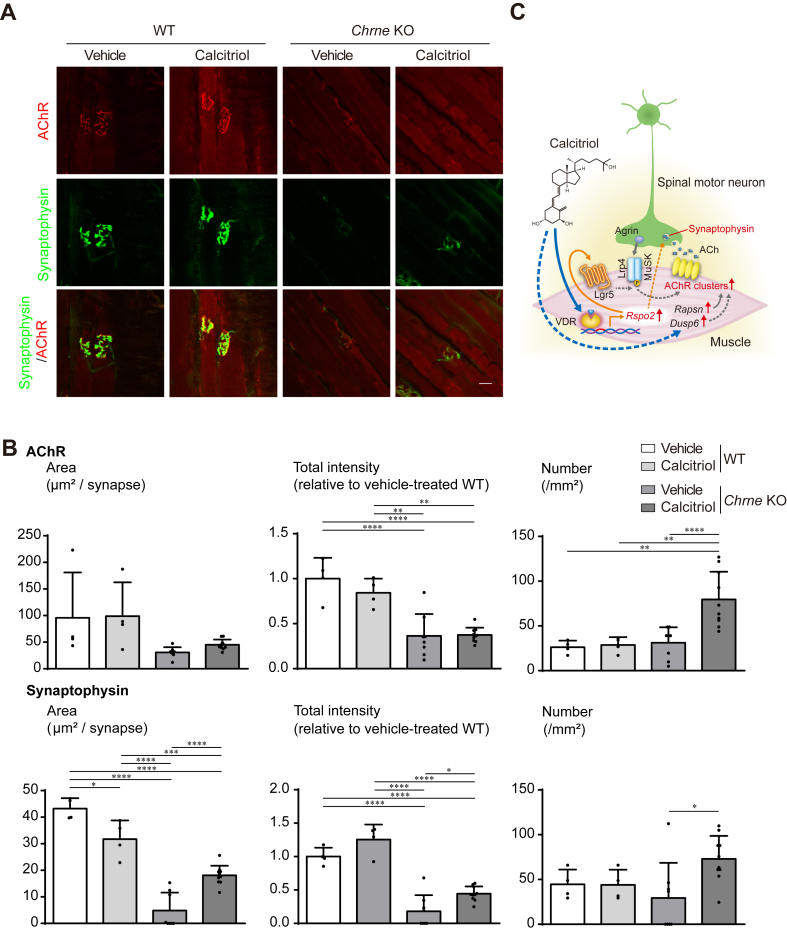
Calcitriol improves the NMJ structure in diaphragm of *Chrne* KO mouse. Wild-type and *Chrne* KO mice were intraperitoneally administrated with 1 ​% ethanol (vehicle) or 0.016 ​μg/kg/day calcitriol in 1 ​% ethanol every day for 2 weeks from 6 to 8 weeks of age. **(A)** Representative confocal images of the NMJs of the right diaphragm at 8 weeks of age stained with anti-synaptophysin antibody (green) and α-bungarotoxin (red) to visualize the nerve terminals and acetylcholine receptors (AChRs), respectively. Scale bar ​= ​25 ​μm. **(B)** Morphometric analyses of the area, total signal intensity, and the number of α-bungarotoxin-positive signals for AChR clusters and synaptophysin-positive signals for synaptic vesicles. AChR areas and synaptophysin-positive areas were manually traced individually and measured by MetaMorph software. The measurement was blinded. Mean ​± ​SD (*n* ​= ​8 NMJs per mouse ​× ​4 mice) are indicated. ∗*p* ​< ​0.05, ∗∗*p* ​< ​0.01, ∗∗∗*p* ​< ​0.001, and ∗∗∗∗*p* ​< ​0.0001 by one-way ANOVA followed by Tukey’s posthoc test. **(C)** Schematic summary of the effect of calcitriol on the NMJ. Calcitriol directly enhances the expression of Rspo2 via VDR. Rspo2 binds to Lgr5 and enhances agrin-LRP4-MuSK signaling pathway [26,27]. Calcitriol markedly increases synaptic vesicles likely via Rspo2. Solid and broken lines indicate direct and indirect effects, respectively.

The molecular effects of calcitriol treatment for 2 weeks were analyzed by qRT-PCR of the diaphragm of *Chrne* KO mice at age 8 weeks. When vehicle-treated wild-type mice and vehicle-treated *Chrne* KO mice were compared, *Chrne* KO mice showed increased *Chrng* and *Rspo2*, which was likely to compensate for loss of *Chrne*, and markedly decreased *Lrp4* (Fig. 4E). The expression level of *Lrp4* was not analyzed in two previously generated *Chrne* KO mouse lines [34,35], and the underling mechanisms remain elusive. The expressions of NMJ-specific genes (*Chrng, Musk*, *Lrp4*, *Colq*, and *Ache*) remained unchanged by calcitriol treatment in *Chrne* KO mice (Fig. 4E). In contrast, the expressions of *Rspo2, Rapsn,* and *Dusp6* were increased, while the expression of the other Wnt activators including *Ctnnb1*, *Lgr5*, and *Axin2,* and *Erbb2* were not. Immunohistochemistry of the diaphragm showed that postsynaptic AChR signals and presynaptic synaptophysin signals were decreased in *Chrne* KO mice (Fig. 5A). Calcitriol markedly increased the number of AChR clusters, as well as the area, the intensity, and the number of synaptic vesicles, in *Chrne* KO mice (Fig. 5B). Taken together, the mouse studies suggested that calcitriol mitigated post- and pre-synaptic abnormalities at the NMJ in *Chrne* KO mice, and extended the mouse survival by improving motor performances, which was likely to be accounted for by the induction of *Rspo2, Rapsn,* and *Dusp6*.

## Discussion

Calcitriol upregulates calcium concentration in blood, which is derived from the intestine, the kidney, and the bone [1]. In myoblasts, calcitriol enhances myotube differentiation by altering the expressions of myogenic regulatory factors [46]. In addition, calcitriol along with insulin upregulates signals for protein biosynthesis in myotubes [47]. A recent report showed that calcitriol enhanced rapsyn expression and agrin-induced AChR clustering in cultured myotubes [18], which prompted us to explore its underlying mechanisms. Here we found that calcitriol increased AChR clustering in C2C12 myotubes, as well as in co-cultured C2C12 myotubes and NSC34 neuronal cells (Fig. 1). In addition, calcitriol upregulated muscle contraction-related genes and downregulated cell division-related genes (Fig. 2), which was likely to represent the induction of myotube differentiation. The effects of calcitriol on the co-cultured cells and the *Chrne* KO mice may be partly accounted for by the induction of myotube differentiation and some other additional effects. In accordance with our observation, knockdown of *Vdr* in C2C12 myoblasts suppresses myotube differentiation [48]. In *Chrne* KO mice, calcitriol ameliorated structural abnormalities of the NMJ and improved motor performances, which culminated in prolonged survival of *Chrne* KO mice (Fig. 4). Adverse reactions of calcitriol in human mostly stem from hypercalcemia, which can be rapidly normalized by terminating calcitriol. We propose that calcitriol is one of potential therapeutic agents for CMS.

ChIP-seq [39] showed that VDR binds to a region approximately 15 ​kbp upstream to *Rspo2* in calcitriol-treated osteoblasts (Fig. 3C). Biallelic deletion of the VDR-binding sites of *Rspo2* in C2C12 myoblasts/myotubes markedly decreased the expression of Rspo2 (Fig. 3E). However, no other *cis*-acting elements were present in the deleted region in either the ENCODE registry of candidate *cis*-regulatory elements (cCREs) [49] or the NCBI RefSeq functional elements [50]. Thus the reason for the marked reduction in the *Rspo2* expression remain undermined. In addition, the biallelic deletion of the VDR-binding sites nullified the calcitriol-induced expression of *Rspo2*, while calcitriol-induced expression of *Rapsn* was preserved (Fig. 3E). We previously showed that secreted Rspo2 binds to its receptor, Lgr5, on the motor endplate and activates the agrin-LRP4-MuSK signaling pathway to enhance AChR clustering [26,27]. This Rspo2 pathway was likely to be facilitated by calcitriol (Fig. 5C). In accordance with our current studies, *Vdr* knockout mice showed small and simplified AChR clusters at the extensor digitorum longus muscle [17], which was likely to be accounted for by lack of VDR-induced enhancement of *Rspo2* expression. RNA-seq analysis of calcitriol-treated co-cultured cells (Fig. 2C), as well as qRT-PCR of C2C12 myotubes (Fig. 3B) and the diaphragm of *Chrne* KO mice (Fig. 4E), revealed that calcitriol upregulated expressions of *Rapsn* and *Dusp6* in addition to *Rspo2*. In contrast to *Rspo2*, ChIP-seq showed no VDR-binding sites for *Rapsn* or *Dusp6* [39], and the calcitriol-mediated induction of *Rapsn* and *Dusp6* were canceled by cycloheximide. Thus, calcitriol was likely to indirectly induce the expressions of *Rapsn* or *Dusp6.* Indeed, we previously showed that Rspo2 increased the expression of rapsyn protein in C2C12 myotubes [26]. Calcitriol-mediated enhancement of rapsyn expression in C2C12 myotubes [18] was also likely to be driven by Rspo2 expression. In contrast to *Rspo2* and *Rapsn*, the mechanisms of the calcitriol-mediated induction of *Dusp6* remain elusive. Dual-specificity phosphatase 6 (Dusp6) is a 42-kDa cytoplasmic protein that dephosphorylates both tyrosine and serine/threonine residues of growth factor-activated MAPKs but not stress-induced MAPKs [51,52]. Laser capture microdissection of the mouse NMJ showed that *Dusp6* was 36.1-times more expressed at the NMJ compared to the extrajunctional region, and was the top 13th gene enriched at the NMJ [53]. NMJ-specific expressions of *Dusp6* mRNA and Dusp6 protein were also confirmed by *in situ* hybridization and immunostaining, respectively [43]. Dusp6 is a genetic modifier of muscular dystrophy in *Sgcg*-deficient D2 mouse [54]. D2 mouse carries p.Met62Ile in Dusp6, which reduces the binding of Dusp6 to Erk2 and subsequently increases Erk1/Erk2 phosphorylation. Inhibition of Dusp6 in myoblasts isolated from wild-type mice, but not from D2 mice, increases cell proliferation [54]. Thus, Dusp6 may enhance the differentiation of specific unidentified cells at the motor endplate. Further mechanistic analyses are required to elucidate the exact roles of calcitriol on Dusp6 at the NMJ.

## Author Contributions

BO, SI, and KO conceived the study. HT performed cellular studies with the help of BO, TI, SK, and HK. BO performed animal studies with the help of HT. HN, JT, and TOg performed RNA-seq analysis. SZ established R2-VDR KO C2C12 ​cell line. YM and TOh established a mouse line lacking *Chrne*. HN, MI, and AM supervised each experiment. NI, SI, TOg, and KO supervised the project and provided financial supports. BO, HT, and KO prepared the manuscript and all authors critically reviewed the contents.

## Data Availability

The authors confirm that the data supporting the findings of this study are available within the article and its supplementary material. The RNA-seq data are available in the DNA Data Bank of Japan (DDBJ) with the accession number of DRA016803.

## Declaration of Competing Interest

The authors declare that they have no known competing financial interests or personal relationships that could have appeared to influence the work reported in this paper.

## Appendix A Supplementary data

Figure S1 Seeking for an optimal dose of calcitriol to enhance AChR clustering in C2C12 myotubes cultured alone (A) and co-cultured with NSC34 ​cells (B) Culturing protocols of C2C12 myoblasts/myotubes alone and with NSC34 ​cells are indicated in Fig. 1A and B, respectively. Total area, total intensity, and total length of AChR clusters were blindly measured using MetaMorph software, and values were normalized to that without calcitriol. Mean and SD are indicated (*n* ​= ​6 visual fields per well ​× ​4 wells). ∗*p* ​< ​0.05, ∗∗*p* ​< ​0.01,∗∗∗*p* ​< ​0.001, and ∗∗∗∗*p* ​< ​0.0001 by one-way ANOVA followed by Tukey’s posthoc test. Figure S1

Figure S2 RNA-seq analysis of co-cultured NSC34 motor neurons and C2C12 myotubes with or without 10^−10^ ​M calcitriol (*n* ​= ​3 wells each). (A) Volcano plot showing the effect of 10^−10^ ​M calcitriol. Thresholds of fold change and false discovery rate were arbitrarily set to select the top 30 upregulated genes. The top 30 upregulated genes are indicated in Fig. 2A. *Slc26a8* encoding a transporter for electroneutral exchange of chloride for bicarbonate or sulfate across the plasma membrane was the only gene that was downregulated. (B) The enrichment plot of “striated muscle contraction” by GSEA. Heatmap of the included genes is shown in Fig. 2B. Figure S2

Figure S3 ChIP-seq of VDR and biallelic deletion of the VDR-binding site in the distal promoter region of *Rspo2* in C2C12 ​cells. **(A)** ChIP-seq tracks of VDR of *Cdon* and *Rspo2* in vehicle- and calcitriol-treated differentiated IDG-SW3 cells [39]. ChIP-seq reads were normalized to 10^7^ tags. ChIP-seq peaks of VDR (arrowhead) are indicated in blue letters. gRNA targets are indicated by boxes. A 633-bp deletion (chr15: 43,179,529–43,180,161 on GRCm38/mm10) and a 631-bp deletion (chr15: 43,179,529–43,180,159 on GRCm38/mm10) in R2-VDR KO C2C12 ​cells are indicated by red underlines. **(B)** Representative images showing the shapes of control and R2-VDR KO C2C12 myotubes. Bar ​= ​200 ​μm. **(C)** Representative images of AChR clustering with agrin (5 ​ng/ml) and calcitriol (10^−10^ ​M) in wild-type (WT) and R2-VDR KO C2C12 myotubes. Bar ​= ​10 ​μm. AChR areas were blindly measured by MetaMorph software. Mean ​± ​SD (*n* ​= ​5 visual fields per well ​× ​3 wells) are indicated. ∗∗∗∗*p* ​< ​0.001 by one-way ANOVA followed by Tukey’s posthoc test. Figure S3

Figure S4 Calcitriol administration has no effects on body weight or muscle weights, and improves motor functions in *Chrne* KO mice. **(A)** Body weights of *Chrne* KO mice administered with indicated amounts of calcitriol. Mean and SD are indicated. No statistical difference by two-way ANOVA. **(B)** Temporal profile of body weights of wild-type (WT) and *Chrne* KO mice with or without 0.016 ​μg/kg/day calcitriol for 2 weeks (*n* ​= ​2 to 11 mice each). Mean and SD are indicated. No statistical difference between vehicle- and calcitriol-treated mice (*p* ​= ​0.327 and 0.744 for WT and *Chrne* KO mice, respectively) by two-way repeated measures ANOVA. **(C)** Wet weights of quadriceps femoris, gastrocnemius, and tibialis anterior muscles in *Chrne* KO mice with or without calcitriol for 2 weeks (*n* ​= ​4 mice each). The summed weights of bilateral muscles are indicated. Mean and SD are indicated. No statistical significance by one-way ANOVA followed by Sidak’s posthoc test. **(D**–**G)** Endurance time on a rota-rod **(D, F)** and the number of crossing beams per hour by voluntary exercise test **(E, G)** of wild-type (WT) and *Chrne* KO mice with or without calcitriol (*n* ​= ​3 to 6 mice each). **F** and **G** show the results of *Chrne* KO mice only. ∗*p* ​< ​0.05 and ∗∗∗*p* ​< ​0.001 two-way repeated measures ANOVA followed by Sidak’s posthoc test. Asterisks are indicated only between vehicle- and calcitriol-treated mice, and not between WT and *Chrne* KO mice. Figure S4
