# Supplementary figures and images for: Calcitriol ameliorates motor deficits and prolongs survival of Chrne-deficient mouse, a model for congenital myasthenic syndrome, by inducing Rspo2
Source: Neurotherapeutics. 2024 Jan 16;21(2):e00318. doi: 10.1016/j.neurot.2024.e00318 (PMC10963930; doi:10.1016/j.neurot.2024.e00318)

**A**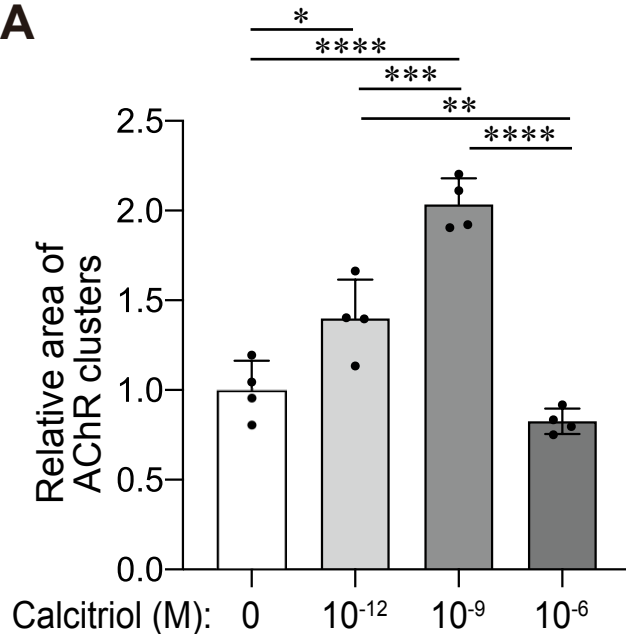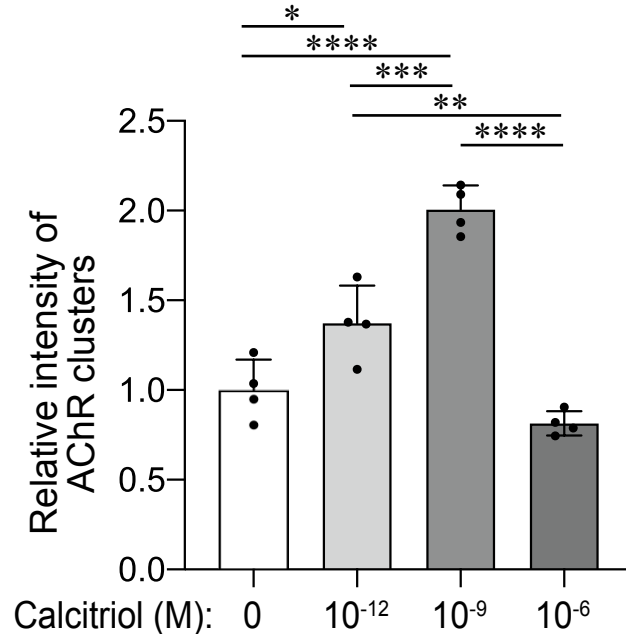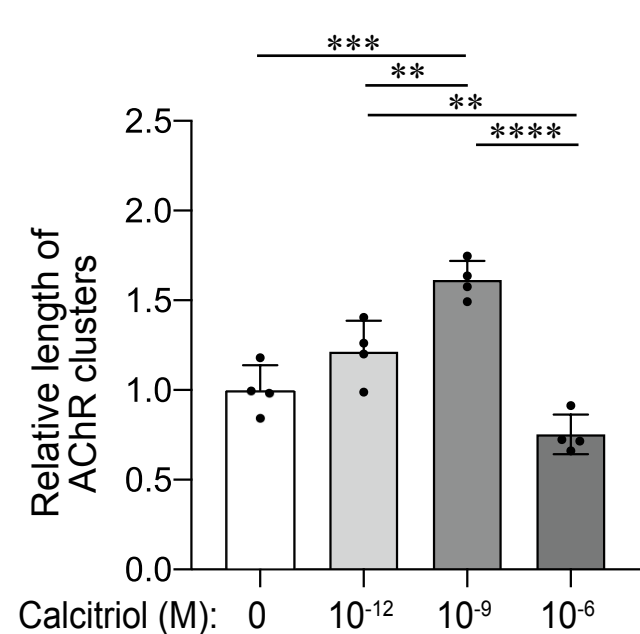**B**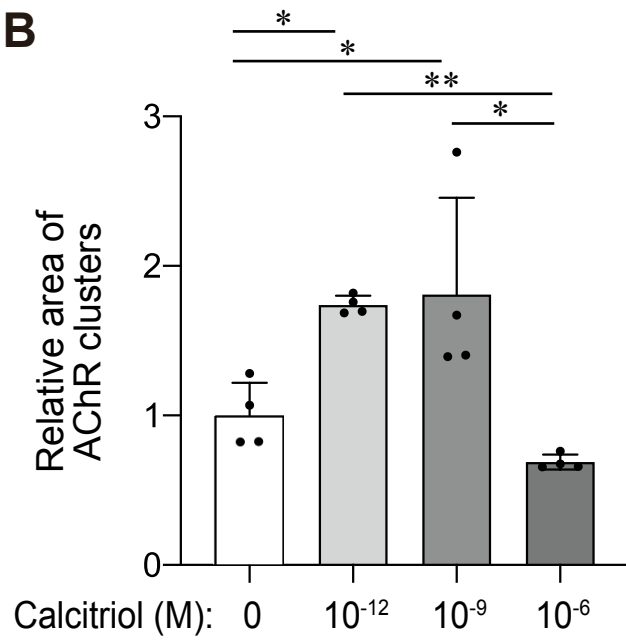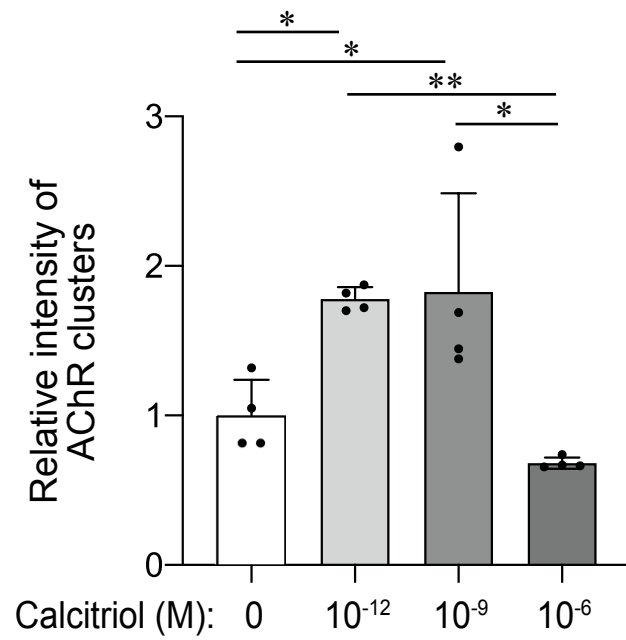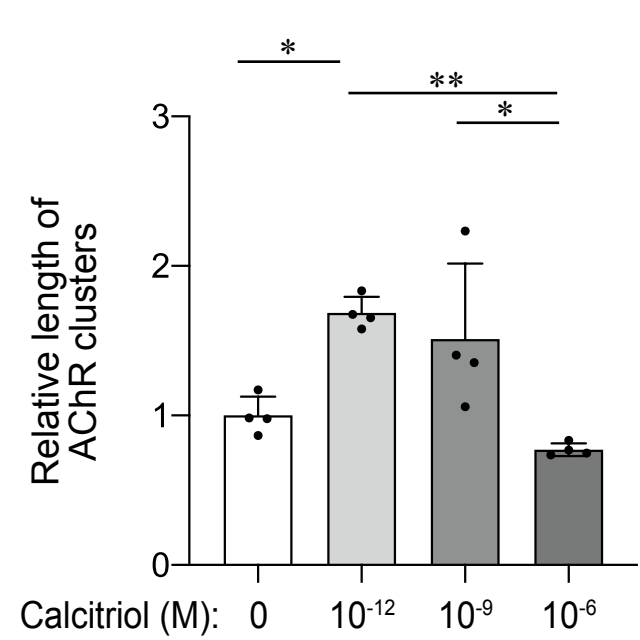

Supplement: Figure S1 — Seeking for an optimal dose of calcitriol to enhance AChR clustering in C2C12 myotubes cultured alone (A) and co-cultured with NSC34 ​cells (B) Culturing protocols of C2C12 myoblasts/myotubes alone and with NSC34 ​cells are indicated in Fig. 1A and B, respectively. Total area, total intensity, and total length of AChR clusters were blindly measured using MetaMorph software, and values were normalized to that without calcitriol. Mean and SD are indicated (n ​= ​6 visual fields per well ​× ​4 wells). ∗p ​< ​0.05, ∗∗p ​< ​0.01,∗∗∗p ​< ​0.001, and ∗∗∗∗p ​< ​0.0001 by one-way ANOVA followed by Tukey’s posthoc test. [file mmc1.pdf]

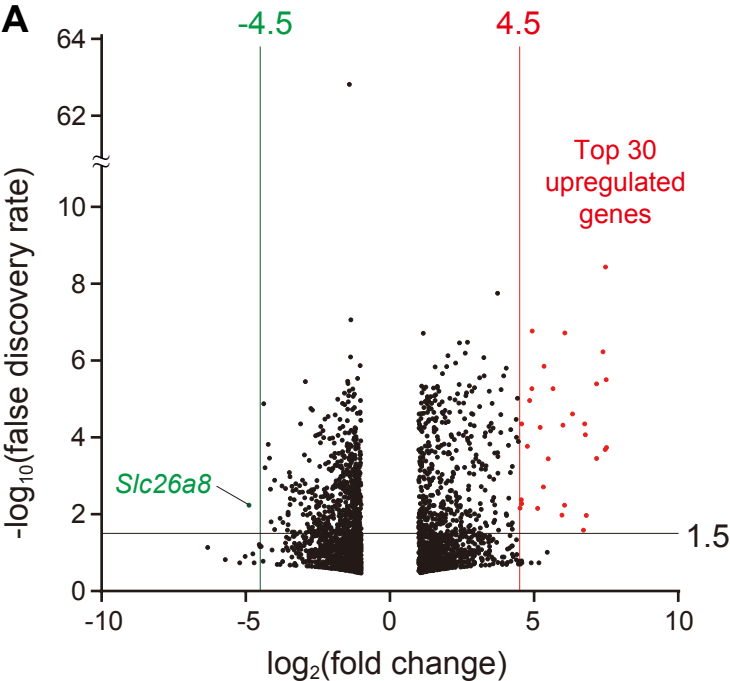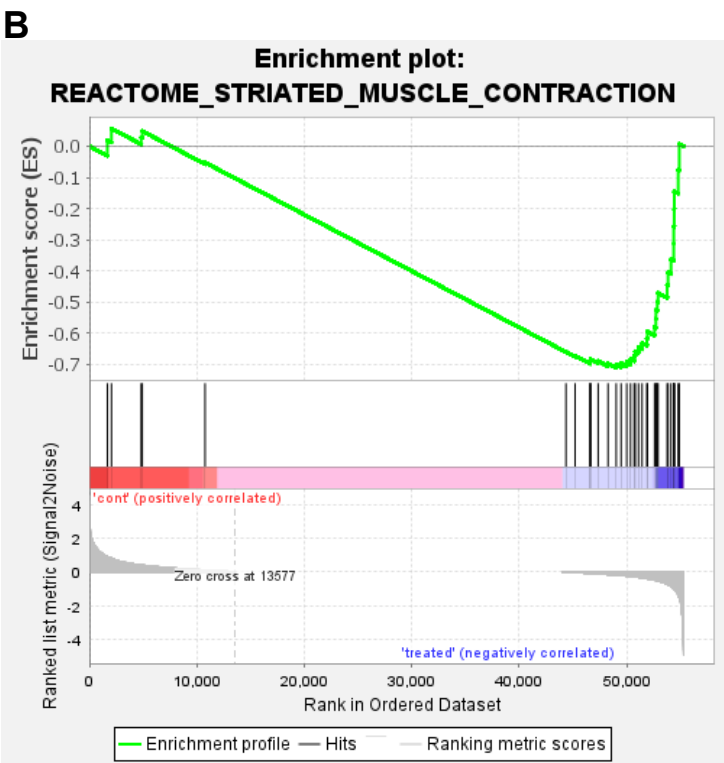

Supplement: Figure S2 — RNA-seq analysis of co-cultured NSC34 motor neurons and C2C12 myotubes with or without 10−10 ​M calcitriol (n ​= ​3 wells each). (A) Volcano plot showing the effect of 10−10 ​M calcitriol. Thresholds of fold change and false discovery rate were arbitrarily set to select the top 30 upregulated genes. The top 30 upregulated genes are indicated in Fig. 2A. Slc26a8 encoding a transporter for electroneutral exchange of chloride for bicarbonate or sulfate across the plasma membrane was the only gene that was downregulated. (B) The enrichment plot of “striated muscle contraction” by GSEA. Heatmap of the included genes is shown in Fig. 2B. [file mmc2.pdf]

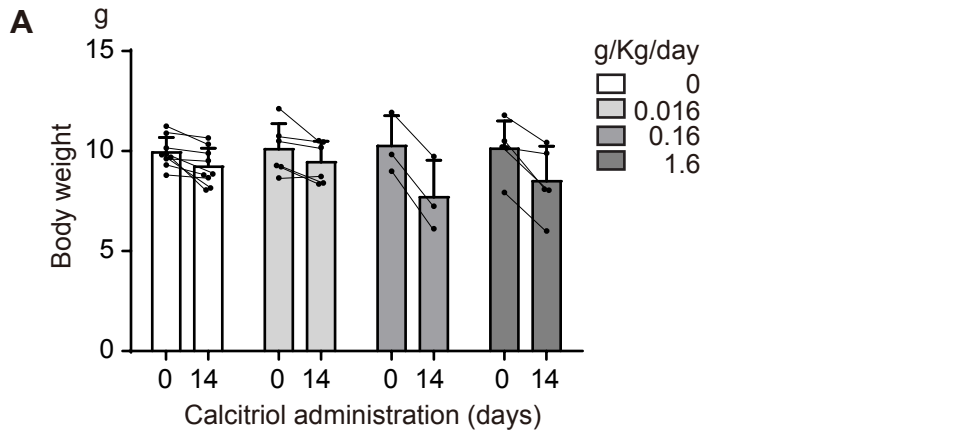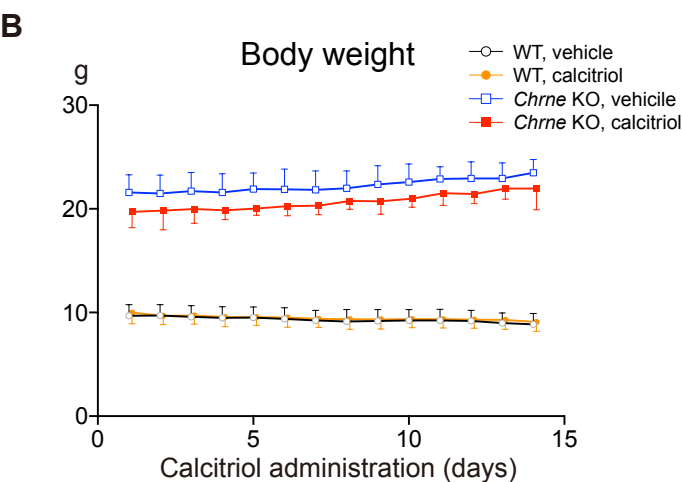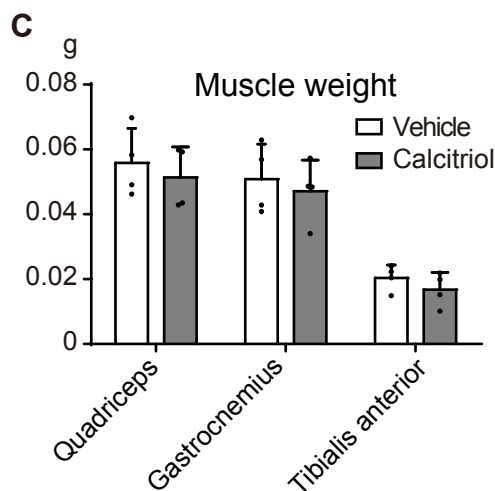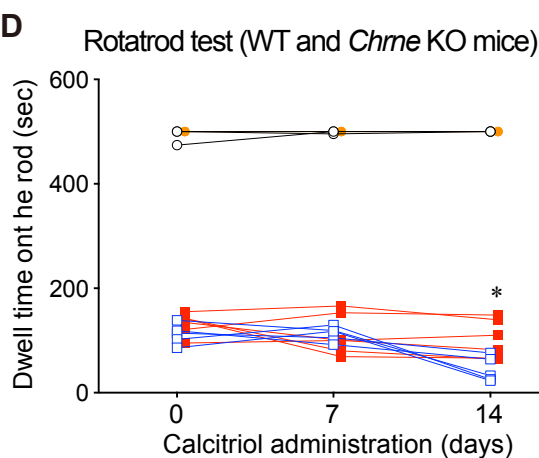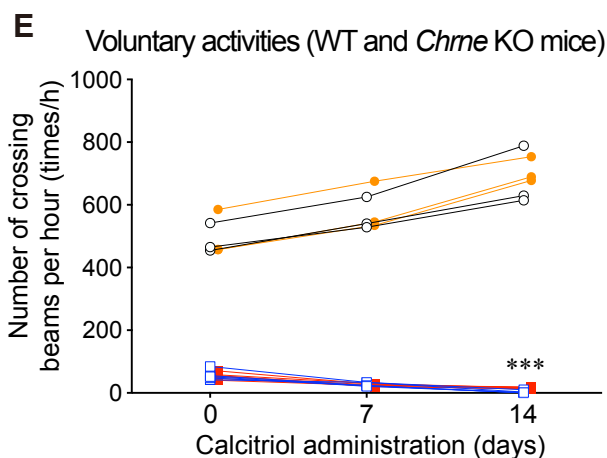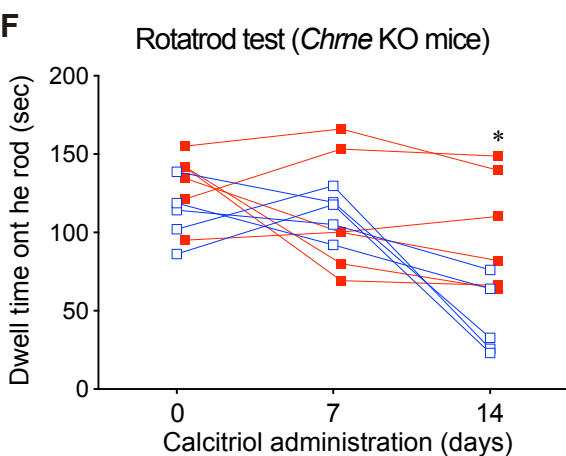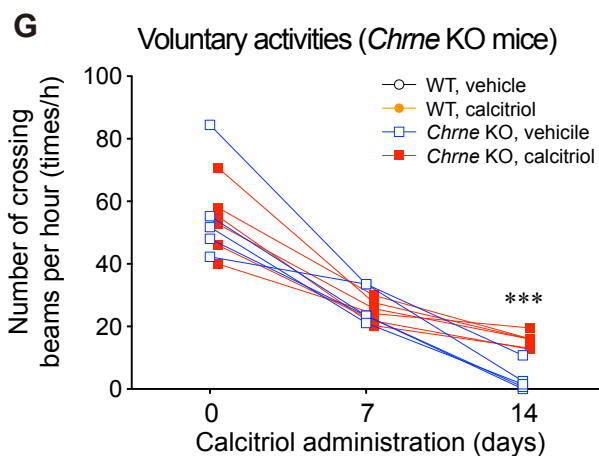

Supplement: Figure S4 — Calcitriol administration has no effects on body weight or muscle weights, and improves motor functions in Chrne KO mice. (A) Body weights of Chrne KO mice administered with indicated amounts of calcitriol. Mean and SD are indicated. No statistical difference by two-way ANOVA. (B) Temporal profile of body weights of wild-type (WT) and Chrne KO mice with or without 0.016 ​μg/kg/day calcitriol for 2 weeks (n ​= ​2 to 11 mice each). Mean and SD are indicated. No statistical difference between vehicle- and calcitriol-treated mice (p ​= ​0.327 and 0.744 for WT and Chrne KO mice, respectively) by two-way repeated measures ANOVA. (C) Wet weights of quadriceps femoris, gastrocnemius, and tibialis anterior muscles in Chrne KO mice with or without calcitriol for 2 weeks (n ​= ​4 mice each). The summed weights of bilateral muscles are indicated. Mean and SD are indicated. No statistical significance by one-way ANOVA followed by Sidak’s posthoc test. (D–G) Endurance time on a rota-rod (D, F) and the number of crossing beams per hour by voluntary exercise test (E, G) of wild-type (WT) and Chrne KO mice with or without calcitriol (n ​= ​3 to 6 mice each). F and G show the results of Chrne KO mice only. ∗p ​< ​0.05 and ∗∗∗p ​< ​0.001 two-way repeated measures ANOVA followed by Sidak’s posthoc test. Asterisks are indicated only between vehicle- and calcitriol-treated mice, and not between WT and Chrne KO mice. [file mmc4.pdf]
