# Supplementary material for: Calcitriol ameliorates motor deficits and prolongs survival of Chrne-deficient mouse, a model for congenital myasthenic syndrome, by inducing Rspo2
Source: Neurotherapeutics. 2024 Jan 16;21(2):e00318. doi: 10.1016/j.neurot.2024.e00318 (PMC10963930; doi:10.1016/j.neurot.2024.e00318)
Supplement: Figure S3 — ChIP-seq of VDR and biallelic deletion of the VDR-binding site in the distal promoter region of Rspo2 in C2C12 ​cells. (A) ChIP-seq tracks of VDR of Cdon and Rspo2 in vehicle- and calcitriol-treated differentiated IDG-SW3 cells [39]. ChIP-seq reads were normalized to 107 tags. ChIP-seq peaks of VDR (arrowhead) are indicated in blue letters. gRNA targets are indicated by boxes. A 633-bp deletion (chr15: 43,179,529–43,180,161 on GRCm38/mm10) and a 631-bp deletion (chr15: 43,179,529–43,180,159 on GRCm38/mm10) in R2-VDR KO C2C12 ​cells are indicated by red underlines. (B) Representative images showing the shapes of control and R2-VDR KO C2C12 myotubes. Bar ​= ​200 ​μm. (C) Representative images of AChR clustering with agrin (5 ​ng/ml) and calcitriol (10−10 ​M) in wild-type (WT) and R2-VDR KO C2C12 myotubes. Bar ​= ​10 ​μm. AChR areas were blindly measured by MetaMorph software. Mean ​± ​SD (n ​= ​5 visual fields per well ​× ​3 wells) are indicated. ∗∗∗∗p ​< ​0.001 by one-way ANOVA followed by Tukey’s posthoc test. [file mmc3.pdf]

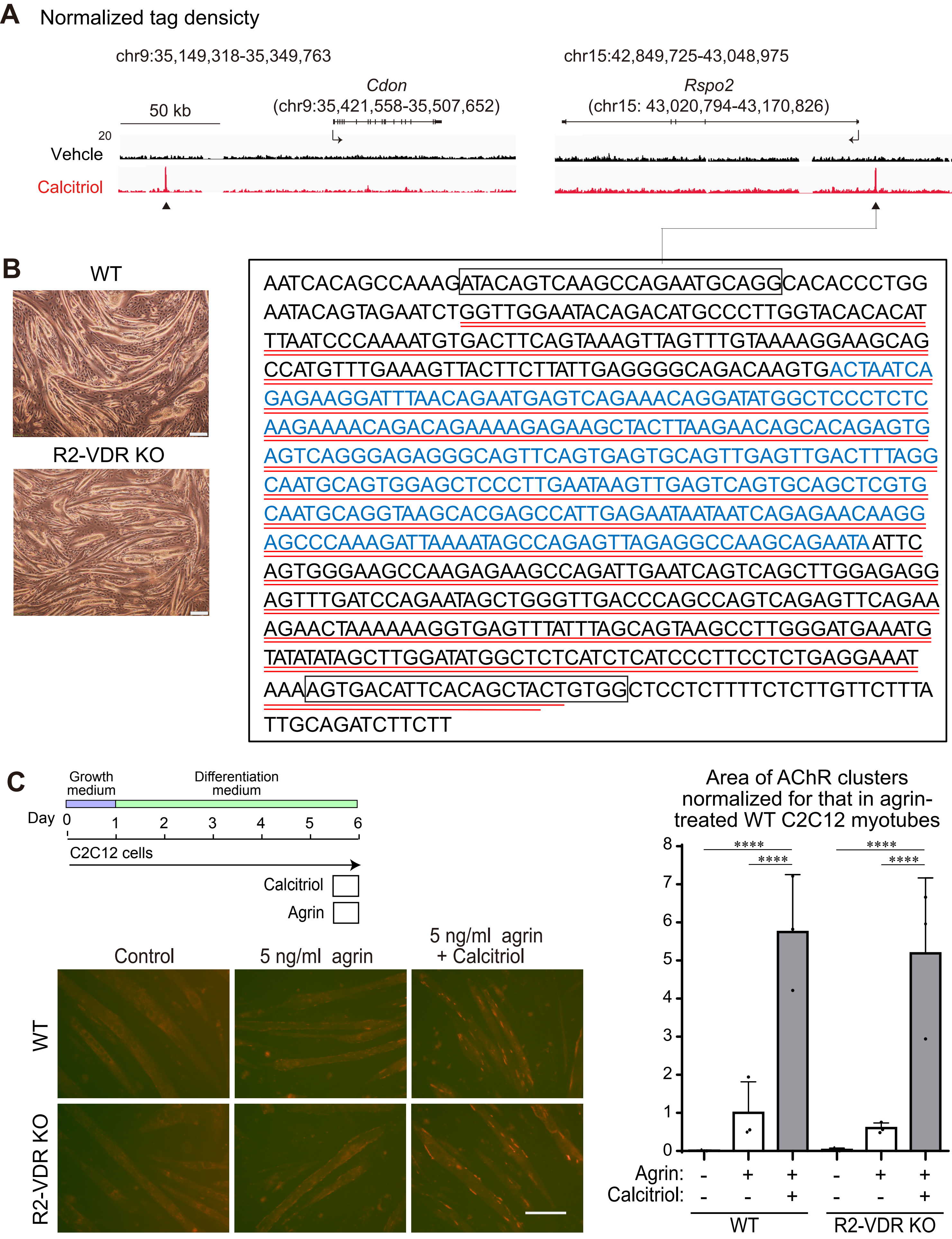
A
Normalized tag densicty
chr9:35,149,318-35,349,763
Cdon
(chr9:35,421,558-35,507,652)
50 kb
20
Vehcle
Calcitriol
chr15:42,849,725-43,048,975
Rspo2
(chr15: 43,020,794-43,170,826)
B
WT
R2-VDR KO
AATCACAGCCAAAGATACAGTCAAGCCAGAATGCAGGCACACCCTGG
AATACAGTAGAATCTGGTTGGAATACAGACATGCCCTTGGTACACACAT
TTAATCCCAAAATGTGACTTCAGTAAAGTTAGTTTGTAAAAGGAAGCAG
CCATGTTTGAAAGTTACTTCTTATTGAGGGGCAGACAAGTGACTAATCA
GAGAAGGATTTAACAGAATGAGTCAGAAACAGGATATGGCTCCCTCTC
AAGAAAACAGACAGAAAAGAGAAGCTACTTAAGAACAGCACAGAGTG
AGTCAGGGAGAGGGCAGTTCAGTGAGTGCAGTTGAGTTGACTTTAGG
CAATGCAGTGGAGCTCCCTTGAATAAGTTGAGTCAGTGCAGCTCGTG
CAATGCAGGTAAGCACGAGCCATTGAGAATAATAATCAGAGAACAAGG
AGCCCAAAGATTAAAATAGCCAGAGTTAGAGGCCAAGCAGAATAATTC
AGTGGGAAGCCAAGAGAAGCCAGATTGAATCAGTCAGCTTGGAGAGG
AGTTTGATCCAGAATAGCTGGGTTGACCCAGCCAGTCAGAGTTCAGAA
AGAACTAAAAAAGGTGAGTTTATTTAGCAGTAAGCCTTGGGATGAAATG
TATATATAGCTTGGATATGGCTCTCATCTCATCCCTTCCTCTGAGGAAAT
AAAAGTGACATTCACAGCTACTGTGGCTCCTCTTTTCTCTTGTTCTTTA
TTGCAGATCTTCTT
C
Growth medium
Differentiation medium
Day 0 1 2 3 4 5 6
C2C12 cells
Calcitriol
Agrin
Control
5 ng/ml agrin
5 ng/ml agrin + Calcitriol
WT
R2-VDR KO
Area of AChR clusters normalized for that in agrin-treated WT C2C12 myotubes
0 1 2 3 4 5 6 7 8
****
****
****
****
Agrin: - + + - + +
Calcitriol: - - + - - +
WT
R2-VDR KO
