# Supplementary material for: Calcitriol ameliorates motor deficits and prolongs survival of Chrne-deficient mouse, a model for congenital myasthenic syndrome, by inducing Rspo2
Source: Neurotherapeutics. 2024 Jan 16;21(2):e00318. doi: 10.1016/j.neurot.2024.e00318 (PMC10963930; doi:10.1016/j.neurot.2024.e00318)
Supplement: Multimedia component 5 [file mmc5.docx]

**Supplementary Table S1. Reactome gene sets increased in calcitriol-treated *in vitro* NMJ**

| **Name** | **NES** | ***P*** | **FDR** |
| --- | --- | --- | --- |
| COMPLEMENT_CASCADE | 1.573 | 0.005 | 0.576 |
| INITIAL_TRIGGERING_OF_COMPLEMENT | 1.592 | 0.005 | 0.628 |
| RESPIRATORY_ELECTRON_TRANSPORT_ATP_SYNTHESIS_BY_CHEMIOSMOTIC_COUPLING_AND_HEAT_PRODUCTION_BY_UNCOUPLING_PROTEINS | 1.545 | <0.001 | 0.679 |
| RSK_ACTIVATION | 0.456 | 0.998 | 0.999 |
| RESPIRATORY_ELECTRON_TRANSPORT | 1.594 | <0.001 | 1.000 |
| VITAMIN_B5_PANTOTHENATE_METABOLISM | 1.500 | 0.021 | 1.000 |
| THE_CITRIC_ACID_TCA_CYCLE_AND_RESPIRATORY_ELECTRON_TRANSPORT | 1.492 | <0.001 | 1.000 |
| PEROXISOMAL_LIPID_METABOLISM | 1.459 | 0.033 | 1.000 |
| STRIATED_MUSCLE_CONTRACTION | 1.449 | 0.033 | 1.000 |

Top ten Reactome gene sets are indicated in ascending order of false-discovery rate (FDR) followed by descending order of normalized enrichment score (NES).

**Supplementary Table S2. Reactome gene sets decreased in calcitriol-treated *in vitro* NMJ**

| **Name** | **NES** | ***P*** | **FDR** |
| --- | --- | --- | --- |
| MITOTIC_PROMETAPHASE | -1.936 | <0.001 | <0.001 |
| CELL_CYCLE_CHECKPOINTS | -1.904 | <0.001 | <0.001 |
| RESOLUTION_OF_SISTER_CHROMATID_COHESION | -1.881 | <0.001 | <0.001 |
| MITOTIC_METAPHASE_AND_ANAPHASE | -1.855 | <0.001 | <0.001 |
| M_PHASE | -1.855 | <0.001 | <0.001 |
| MITOTIC_G1_PHASE_AND_G1_S_TRANSITION | -1.840 | <0.001 | <0.001 |
| SEPARATION_OF_SISTER_CHROMATIDS | -1.832 | <0.001 | <0.001 |
| RHO_GTPASES_ACTIVATE_FORMINS | -1.816 | <0.001 | <0.001 |
| MITOTIC_SPINDLE_CHECKPOINT | -1.813 | <0.001 | <0.001 |
| DNA_REPLICATION | -1.801 | <0.001 | <0.001 |
| S_PHASE | -1.797 | <0.001 | <0.001 |
| CHROMOSOME_MAINTENANCE | -1.791 | <0.001 | <0.001 |
| DNA_REPLICATION_PRE_INITIATION | -1.757 | <0.001 | 0.000 |
| G2_M_CHECKPOINTS | -1.759 | <0.001 | 0.000 |
| INTERFERON_ALPHA_BETA_SIGNALING | -1.725 | <0.001 | 0.000 |
| HDR_THROUGH_HOMOLOGOUS_RECOMBINATION_HRR_ | -1.721 | <0.001 | 0.001 |
| HOMOLOGY_DIRECTED_REPAIR | -1.709 | <0.001 | 0.001 |
| EXTENSION_OF_TELOMERES | -1.700 | <0.001 | 0.001 |
| DNA_REPAIR | -1.693 | <0.001 | 0.001 |
| DNA_STRAND_ELONGATION | -1.695 | <0.001 | 0.001 |
| DEPOSITION_OF_NEW_CENPA_CONTAINING_NUCLEOSOMES_AT_THE_CENTROMERE | -1.688 | <0.001 | 0.001 |
| DNA_DOUBLE_STRAND_BREAK_REPAIR | -1.687 | <0.001 | 0.001 |
| MITOTIC_G2_G2_M_PHASES | -1.677 | <0.001 | 0.002 |
| ACTIVATION_OF_THE_PRE_REPLICATIVE_COMPLEX | -1.671 | 0.002 | 0.002 |
| RHO_GTPASE_EFFECTORS | -1.670 | <0.001 | 0.002 |
| TELOMERE_MAINTENANCE | -1.656 | <0.001 | 0.003 |
| ACTIVATION_OF_ATR_IN_RESPONSE_TO_REPLICATION_STRESS | -1.646 | 0.002 | 0.004 |
| NUCLEAR_ENVELOPE_NE_REASSEMBLY | -1.647 | <0.001 | 0.004 |
| ORC1_REMOVAL_FROM_CHROMATIN | -1.637 | <0.001 | 0.005 |
| ASSEMBLY_OF_THE_PRE_REPLICATIVE_COMPLEX | -1.638 | <0.001 | 0.005 |
| HCMV_EARLY_EVENTS | -1.640 | <0.001 | 0.005 |
| HOMOLOGOUS_DNA_PAIRING_AND_STRAND_EXCHANGE | -1.627 | 0.002 | 0.006 |
| MITOTIC_PROPHASE | -1.625 | <0.001 | 0.006 |
| PROCESSING_OF_DNA_DOUBLE_STRAND_BREAK_ENDS | -1.619 | <0.001 | 0.007 |
| G1_S_SPECIFIC_TRANSCRIPTION | -1.619 | <0.001 | 0.007 |
| INTERFERON_SIGNALING | -1.618 | <0.001 | 0.007 |
| TERMINATION_OF_TRANSLESION_DNA_SYNTHESIS | -1.612 | <0.001 | 0.008 |
| G2_M_DNA_DAMAGE_CHECKPOINT | -1.610 | <0.001 | 0.008 |
| INFLUENZA_INFECTION | -1.612 | <0.001 | 0.008 |
| RECRUITMENT_OF_NUMA_TO_MITOTIC_CENTROSOMES | -1.599 | 0.002 | 0.011 |
| MEIOSIS | -1.595 | <0.001 | 0.012 |
| AURKA_ACTIVATION_BY_TPX2 | -1.594 | <0.001 | 0.012 |
| TELOMERE_C_STRAND_LAGGING_STRAND_SYNTHESIS | -1.591 | 0.002 | 0.012 |
| SWITCHING_OF_ORIGINS_TO_A_POST_REPLICATIVE_STATE | -1.591 | <0.001 | 0.012 |
| DNA_DAMAGE_BYPASS | -1.587 | <0.001 | 0.013 |
| PROCESSING_OF_CAPPED_INTRON_CONTAINING_PRE_MRNA | -1.582 | <0.001 | 0.015 |
| SUMOYLATION_OF_DNA_REPLICATION_PROTEINS | -1.579 | 0.003 | 0.016 |
| REGULATION_OF_PLK1_ACTIVITY_AT_G2_M_TRANSITION | -1.572 | <0.001 | 0.019 |
| RECRUITMENT_OF_MITOTIC_CENTROSOME_PROTEINS_AND_COMPLEXES | -1.567 | 0.003 | 0.021 |
| TRANSCRIPTIONAL_REGULATION_BY_SMALL_RNAS | -1.566 | 0.003 | 0.021 |
| RESOLUTION_OF_D_LOOP_STRUCTURES | -1.567 | 0.005 | 0.021 |
| G0_AND_EARLY_G1 | -1.557 | <0.001 | 0.024 |
| MEIOTIC_RECOMBINATION | -1.558 | <0.001 | 0.025 |
| MRNA_SPLICING | -1.555 | <0.001 | 0.025 |
| APC_C_MEDIATED_DEGRADATION_OF_CELL_CYCLE_PROTEINS | -1.555 | <0.001 | 0.025 |
| SIGNALING_BY_RHO_GTPASES | -1.550 | <0.001 | 0.027 |
| RESOLUTION_OF_D_LOOP_STRUCTURES_THROUGH_SYNTHESIS_DEPENDENT_STRAND_ANNEALING_SDSA_ | -1.549 | 0.002 | 0.027 |
| TRANSLESION_SYNTHESIS_BY_Y_FAMILY_DNA_POLYMERASES_BYPASSES_LESIONS_ON_DNA_TEMPLATE | -1.550 | 0.005 | 0.027 |
| SCF_SKP2_MEDIATED_DEGRADATION_OF_P27_P21 | -1.545 | 0.003 | 0.029 |
| LAGGING_STRAND_SYNTHESIS | -1.540 | 0.002 | 0.030 |
| BASE_EXCISION_REPAIR | -1.539 | 0.003 | 0.031 |
| RECOGNITION_OF_DNA_DAMAGE_BY_PCNA_CONTAINING_REPLICATION_COMPLEX | -1.540 | 0.003 | 0.031 |
| CONDENSATION_OF_PROPHASE_CHROMOSOMES | -1.541 | 0.006 | 0.031 |
| GAP_FILLING_DNA_REPAIR_SYNTHESIS_AND_LIGATION_IN_GG_NER | -1.533 | 0.007 | 0.035 |
| REGULATION_OF_TP53_ACTIVITY_THROUGH_PHOSPHORYLATION | -1.532 | 0.002 | 0.035 |
| REGULATION_OF_TP53_ACTIVITY | -1.527 | <0.001 | 0.037 |
| POLYMERASE_SWITCHING_ON_THE_C_STRAND_OF_THE_TELOMERE | -1.527 | 0.003 | 0.037 |
| ANTIVIRAL_MECHANISM_BY_IFN_STIMULATED_GENES | -1.528 | <0.001 | 0.037 |
| SNRNP_ASSEMBLY | -1.524 | 0.006 | 0.038 |
| CYCLIN_A_B1_B2_ASSOCIATED_EVENTS_DURING_G2_M_TRANSITION | -1.524 | 0.003 | 0.038 |
| ANCHORING_OF_THE_BASAL_BODY_TO_THE_PLASMA_MEMBRANE | -1.513 | 0.002 | 0.047 |
| INTERFERON_GAMMA_SIGNALING | -1.510 | 0.005 | 0.049 |
| HDR_THROUGH_SINGLE_STRAND_ANNEALING_SSA_ | -1.508 | 0.010 | 0.049 |
| SRP_DEPENDENT_COTRANSLATIONAL_PROTEIN_TARGETING_TO_MEMBRANE | -1.508 | <0.001 | 0.049 |
| NS1_MEDIATED_EFFECTS_ON_HOST_PATHWAYS | -1.509 | 0.007 | 0.050 |

Seventy-five Reactome gene sets with FDR < 0.05 are indicated in ascending order of false-discovery rate (FDR) followed by ascending order of normalized enrichment score (NES).
